# Recursive regularization for inferring gene networks from time-course gene expression profiles

**DOI:** 10.1186/1752-0509-3-41

**Published:** 2009-04-22

**Authors:** Teppei Shimamura, Seiya Imoto, Rui Yamaguchi, André Fujita, Masao Nagasaki, Satoru Miyano

**Affiliations:** 1 Human Genome Center, Institute of Medical Science, University of Tokyo, 4-6-1 Shirokanedai, Minato-ku, Tokyo 108-8639, Japan

## Abstract

**Background:**

Inferring gene networks from time-course microarray experiments with vector autoregressive (VAR) model is the process of identifying functional associations between genes through multivariate time series. This problem can be cast as a variable selection problem in Statistics. One of the promising methods for variable selection is the elastic net proposed by Zou and Hastie (2005). However, VAR modeling with the elastic net succeeds in increasing the number of true positives while it also results in increasing the number of false positives.

**Results:**

By incorporating relative importance of the VAR coefficients into the elastic net, we propose a new class of regularization, called recursive elastic net, to increase the capability of the elastic net and estimate gene networks based on the VAR model. The recursive elastic net can reduce the number of false positives gradually by updating the importance. Numerical simulations and comparisons demonstrate that the proposed method succeeds in reducing the number of false positives drastically while keeping the high number of true positives in the network inference and achieves two or more times higher true discovery rate (the proportion of true positives among the selected edges) than the competing methods even when the number of time points is small. We also compared our method with various reverse-engineering algorithms on experimental data of MCF-7 breast cancer cells stimulated with two ErbB ligands, EGF and HRG.

**Conclusion:**

The recursive elastic net is a powerful tool for inferring gene networks from time-course gene expression profiles.

## Background

The inference of gene networks from time-course microarray data can be defined as the process of identifying functional interactions between genes over time. Typically, a gene network is represented by a directed or undirected graph where nodes indicate genes encoded in a given organism of interest and edges represent various functional properties. The elucidation of gene networks has been expected to having an essential role for better understanding of molecular mechanisms and can be useful in the identification of new drug targets [1-5].

In this article, we use vector autoregressive (VAR) model [6,7] to estimate gene networks from time-course microarray data. The process of inferring gene networks based on the VAR model is to choose non-zero coefficients in the coefficient matrix, which can be considered as a problem of statistical model selection, especially as a variable selection problem [8]. Although a variety of variable selection methods have been developed, *e.g.*, best-subset selection [9], subset selection [9] and the lasso [10], these methods often suffer from the following crucial problems due to the limited number of samples (time points) compared with the large number of variables (genes) in time-course microarray data.

1. **High computational cost for model selection**: When the number of variables is *m*, there are *m *× 2^*m *^candidate models in model selection. The best-subset selection is computationally prohibitive when the number of variables is large.

2. **High correlation between variables**: When the number of variables is much larger than the number of samples, two or more variables tend to be highly correlated [11]. In this situation, the coefficient estimates of the subset selection or the lasso may change erratically in response to small changes in the observed data, and thus the resulting models have poor performances [12,13]. What is worse that these methods tend to select only one variable from the highly correlated variables [13] which can lead to reducing the number of true positives in gene network inference.

One solution for the above problems is to use a regularization method called *elastic net *[13] which minimizes a penalized loss function with *l*_1_- and *l*_2_-penalties of the coefficients. Applying an *l*_1_-penalty regularizes the least squares fit and shrinks some coefficients exactly to zero, *i.e.*, achieves automatic variable selection, as the lasso does. Adding of an *l*_2_-penalty to an *l*_1_-penalty encourages a grouping effect so that highly correlated variables will be in or out of the model together. The elastic net is also capable of selecting a set of relevant variables with low computational effort even when the number of variables is much larger than the number of observations with LARS-EN algorithm [13]. However, although VAR modeling with the elastic net succeeds in increasing the number of true positives, it also results in increasing the number of false positives. This is because the elastic net shrinks the same amount of the *l*_1_-penalty to each coefficient without assessing their relative importance.

To increase the capability of the elastic net, *i.e.*, to decrease the number of false positives while keeping the high number of true positives in inferring gene networks based on VAR model, we propose a new class of regularization, called *recursive elastic net*, by incorporating relative importance of the coefficients. The recursive elastic net is a kind of iterative procedures that can reduce the number of false positives gradually by updating the relative importance of each VAR coefficient. The performance of variable selection strongly depends on the regularization parameters of the *l*_1_- and *l*_2_-penalty terms in the recursive elastic net. For selecting these regularization parameters automatically, we derive a modified Bayesian information criterion and a modified bias-corrected Akaike information criterion.

## Methods

### Vector Autoregressive Model

We now consider gene expression data in time-course microarray experiments at *n *time steps *t *∈ {1, 2, ..., *n*}. Let ***y***_*t *_= (*y*_*t*,1_, ..., *y*_*t*, *m*_)*' *be the vector of *m *gene expressions at time step *t*. We assume that ***y***_*t *_at time step *t *is generated from ***y***_*t*-1 _at the previous time step *t *- 1 with a first-order VAR model:

(1)

where ***c ***is a constant vector, ***B ***= [*B*_*ij*_]_1≤*i*, *j*≤*m *_is an *m *× *m *autoregressive coefficient matrix, ***ϵ***_*t *_is assumed to follow an *m*-dimensional multivariate normal distribution *N*(**0**, **Σ**), and the notation  denotes the transpose of the vector ***y***_*t*_. Throughout the paper, we assume that **Σ **is a diagonal matrix, that is, . Note that the coefficient *B*_*ij *_measures the influence that node *i *exerts on node *j *and the nonzero *B*_*ij *_provides a functional connectivity which is related to Granger causality [14]. The Granger causality is a concept widely used in econometrics to analyze the relationships between variables and is based on the intuitive idea that a cause always occurs before its effect. Thus, we can describe a gene network by a directed graph based on the coefficient matrix ***B ***where nodes represent genes and edges show functional connectivities. In the estimated graph, node *i *is linked to node *j *by the edge *i *→ *j *if and only if *B*_*ij *_≠ 0.

For simplicity of explanation, we introduce the following notations:



Since the covariance matrix **Σ **is diagonal, the first order VAR model of these observations can then be considered as *m *linear regression models

(2)

Here, we center each input variable so that there is no intercept in (2) and scale each input variable so that the observed standard deviation is equal to one.

The major objective of our analysis is to estimate the coefficient matrix ***B ***from observations. Especially, our interest is its structures, that is, we want to estimate which elements of each column of ***B ***are zero or nonzero. Let denote the set of indices corresponding the nonzero components of  by . The graph inference problem can be formally stated as the problem of predicting  from the observations. This formulation is equivalent to *m *variable selection problems in linear regression where each component in ***y***_*t *_is the response and ***y***_*t*-1 _is the covariates.

### Recursive Elastic Net

From this section, we focus on a variable selection problem in linear regression model with response *y*_*t*, *j *_and covariates *y*_*t*-1,1_, ..., *y*_*t*-1, *m*_. For notational simplicity, we omit the suffix *j *of ***z***_*j*_, ***β***_*j*_, *λ*_1*j *_and *λ*_2*j*_, and use the notation ***z***, ***β***, *λ*_1 _and *λ*_2_.

To obtain a sparse solution for parameter estimation with high prediction accuracy and encourage a group effect between variables in linear regression, Zou and Hastie [13] proposed the elastic net, which combines both *l*_1_-regularization and *l*_2_-regularization together. Similar to the lasso, due to the property of *l*_1_-penalty, the elastic net performs automatic variable selection. It can also choose highly correlated variables which is due to the benefit of *l*_2_-penalty. The loss function of the elastic net for parameter estimation can be represented by

(3)

where *λ*_1 _and *λ*_2 _are regularization parameters. The naive elastic net estimator is then the minimizer of (3):

(4)

Although the naive elastic net overcomes the limitations of the lasso, Zou and Hastie [13] showed the naive elastic net does not perform satisfactorily in numerical simulations and this deficiency in performance is due to imposing a double shrinkage with *l*_1_- and *l*_2_-penalties. To solve the problem, they proposed the elastic net estimator as a bias-corrected version of the naive elastic net estimator given by:

(5)

However, the elastic net suffers from too many false positives in the variable selection problem for extremely high-dimensional data, which will be illustrated in Simulation Section. This is due to the same amount of shrinkage for all the coefficients in the *l*_1_-penalty. That is, the same regularization parameter *λ*_1 _is used for each coefficient without assessing their relative importance. In a typical setting of linear regression, it has been shown that such an excessive *l*_1_-penalty can affect the consistency of model selection [15-18].

To enable different amounts of shrinkage for each coefficient, we define the following weighted loss function:

(6)

where *λ*_1 _and *λ*_2 _are regularization parameters, and *w*_1_, ..., *w*_*m *_are positive coefficient weights. The weighted elastic net estimator and its bias-corrected estimator are defined as:

(7)

and

(8)

The modification of the *l*_1_-penalty was first proposed by Zou [18]. This type of *l*_1_-regularization was applied to parameter estimation for several statistical models, such as Cox's proportional hazard model [19], least absolute deviation regression model [20], and graphical Gaussian model [21].

We next consider how to set the values of *w*_*k*_. One possible way is to select the weights so that they are inversely proportional to the true coefficients  that is,

(9)

where *L *is some specified large value. If *w*_*k *_is small,  tends to be nonzero. While  tends to be zero if *w*_*k *_is large. By using the weight (9) and appropriate values of the regularization parameters, we can choose the true set of variables, *i.e.*, . However, in many situations, one does not know true coefficients, and thus we need some plug-in estimator instead of the true coefficients. In this article, we propose a new multi-step regularization, called *recursive elastic net*. The proposed method tries to obtain a better estimator than the elastic net estimator by solving the weighted elastic net problem and updating the weights alternatively. The recursive elastic net can be described as:

#### Recursive Elastic Net

1. Set a maximum number of iterations to be *M *and choose an initial estimator  by the naive elastic net (4).

2. For the *l*-th iteration (*l *= 1, ..., *M*), define the coefficient weights by using the (*l *- 1)-th weighted elastic net estimator as follows:



3. Solve the weighted elastic net problem and estimate the coefficient vector:

(10)

4. Repeat Step 2 and Step 3 until a stopping criterion is fulfilled or when the number of iterations attains *M*.

The parameter *δ *> 0 is introduced to avoid that the weights of zero-valued components in  take the value of infinity. From numerical simulations which are not demonstrated, *δ *should be taken near 0. In this article, we set *δ *= 10^-5^. Following Zou and Hastie [13], we can also consider a bias-corrected version of the recursive elastic net by replacing (10) with

(11)

We call the iterative procedure using the elastic net estimator (5) as the initial variable weights and updating the coefficients by the bias-corrected weighted elastic net (11) *corrected recursive elastic net*. Note that our solution with the iterative procedure is a local optimum when initializing some variable weights and we cannot guarantee the convergence of a global minimum. Thus, it is important to choose a suitable starting point for the variable weights. In the recursive elastic net and the corrected recursive elastic net, we propose to initialize with the naive elastic net and the elastic net estimators, (4) and (5), that is, the unweighted solutions of (10) and (11), respectively. In the recursive elastic net and the corrected recursive elastic net, the regularization parameters determine whether or not the *l*-th estimator is better than the (*l *- 1)-th estimator. The selection problem of the regularization parameters will be discussed in the latter section.

### Grouping Effect

In this section, we show that the recursive elastic net estimator can lead to a desirable grouping effect for correlated variables. In the framework of linear regression, the grouping effect can be considered as an effect that the coefficients of highly correlated variables should be similar each other. As discussed in Zou and Hastie [13], the lasso does not have the grouping effect and tends to select one among a correlated set. The following Lemma which is quoted from Zou and Hastie [13] guarantees the grouping effect for the recursive elastic net in the situation with identical variables, since the loss function of the recursive elastic net is strictly convex when *λ*_2 _> 0. The Lemma is given by:

**Lemma 1 ***Assume that ****x***_*i *_= ***x***_*j *_*for i*, *j *∈ {1, ..., *m*} *and **is estimated by (10), then **for any λ*_2 _> 0.

Furthermore, the following theorem provides an upper bound on the difference of the *i*-th and *j*-th coefficients for the *l*-th iteration with the proposed method:

**Theorem 1 ***For given dataset *(***z***, ***X***), *regularization parameters *(*λ*_1_, *λ*_2_) *and variable weights of the l-th iteration *, *suppose that the response ****z ****is centered and the covariate matrix ****X ****is standardized. Let **be the recursive elastic net estimator of the l-th iteration (10). Suppose that two coefficients satisfy **and define*

(12)

then

(13)

*where **and **is the sample correlation.*

The proof of Theorem 1 is given by Appendix. We notice that the grouping effect of the recursive elastic net contributes not only the sample correlation *ρ *but also the difference between the *i*-th and *j*-th coefficients of the (*l *- 1)-th iterations . If ***x***_*i *_and ***x***_*j *_are highly correlated, *i.e.*, *ρ *≐ 1, and the difference  is almost zero, then Theorem 1 says that the difference  is also almost zero.

### Computational Algorithm

In this section, we provide an algorithm for solving the weighted elastic net problem in (6) by modifying the LARS-EN algorithm [13]. The modified algorithm called LARS-WEN can be described as:

#### Algorithm: LARS-WEN

1. Define the (*n *- 1) × *m *design matrix by



2. Solve the elastic net problem by the LARS-EN algorithm:



3. Output the weighted lasso estimator  by



For a fixed *λ*_2_, the LARS-WEN algorithm sequentially updates the locations of the nonzero coefficients and its values, and produces the entire solution path of the weighted elastic net estimator . The R source code is available from the authors upon request. The computational cost of the LARS-WEN algorithm is the same as that of the LARS-EN algorithm except for the treatment of Step 1 and Step 3. If the algorithm for calculating  is stopped after *u *steps, it requires *O*(*u*^3 ^+ *mu*^2^) operations.

### Selection of Regularization Parameters

The regularization parameters play a critical role for determining the performance of variable selection with the recursive elastic net. We now discuss how to choose the values of the regularization parameters *λ*_1_and *λ*_2 _in the recursive elastic net. Traditionally, cross-validation is often used for estimating the predictive error and evaluating different models. For example, leave-one-out cross-validation involves using a single observation from the original sample as the validation data and the remaining observations as the training data, and requires additional calculations for repeating such that each observation in the sample is used once as the validation data. Note that we need to choose two regularization parameters for each variable *j *in the VAR model. Thus, cross-validation can be time-consuming and hardly be applied for the regularization parameter selection in the estimation of the VAR model with the recursive elastic net. In order to choose the two regularization parameters, we use the degrees of freedom as a measure of model complexity. The profile of degrees of freedom clearly shows that how the model complexity is controlled by shrinkage, which helps us to pick an optimal model among all the possible candidates [22]. In recent years, an unbiased estimate of the degrees of freedom for the lasso [22] was proposed. Using the result of Zou *et al. *[22], an unbiased estimate of the degrees of freedom for the recursive elastic net in the *l*-th iteration is given by

(14)

where  denotes the active set of  and the corresponding design matrix is given by



Note that  does not include *λ*_1 _and depends on  and *λ*_2_. Let  and  be the coefficient vector and the active set of the LARS-WEN step *α*, respectively, and *λ*_*α*,1 _be the corresponding *l*_1_-regularization parameter of the step *α*. Hence, we can use  in place of *λ*_*α*,1 _as a tuning parameter.

In our numerical studies, two model selection criteria, Bayesian information criterion (BIC) [23] and bias-corrected Akaike information criterion (AICc) [24,25], are considered. Substituting the number of parameters in BIC and AICc by the degrees of the freedom  we can define a modified BIC and a modified AICc for selecting the regularization parameters as

(15)

(16)

where

(17)

For a given *λ*_2_, we choose the optimal step that gives the smallest BIC or the smallest AICc from the candidate models.

### VAR Modeling with Recursive Elastic Net

As shown in the previous section, the inference of nonzero components in the VAR model can be formalized as *m *variable selection problems, *i.e.*, our objective is to estimate . Therefore, we can apply the recursive elastic net directly to estimate the coefficient matrix ***B ***in the VAR model. In this section, we provide an algorithm for estimating ***B ***in the VAR model with the recursive elastic net and the derived model selection criteria for a fixed *λ*_2_. The algorithm is as follows:

#### Algorithm:RENET-VAR

1. Set a maximum number of iterations to be *M *and a maximum step size to be *u*.

2. For a fixed *λ*_2_, start with the initial coefficient weights



3. For *j *= 1, ..., *m*

(a) For the *l*-th iteration, given , the LARS-WEN algorithm after *u *steps produces *u *solutions,



where

(18)

(b) Calculate the values of model selection criterion for *u *candidate models, say



where  denotes the active set of , and MC = AICc or BIC.

(c) Select  from among  which satisfies



(d) Update the variable weights by



4. Update *l *= *l *+ 1 and repeat Step 3 until a stopping criterion is satisfied or the number of iterations attains *M*.

As a stopping criterion, we calculate the sum of the values of model selection criterion for *j *= 1, ..., *m*:

(19)

The algorithm stops if SMC^(*l*) ^(*λ*_2_) - SMC^(*l *- 1)^(*λ*_2_) > 0 (*l *= 2, 3, ..., *M*) holds.

Typically, we first pick a grid of values for *λ*_2_, say



For each *λ*_*γ*, 2_, the RENET-VAR algorithm produces . Finally, we select  which satisfies



We can also estimate  with the bias-corrected recursive elastic net by replacing the solution (18) with



We note that our iterative algorithm guarantees the decrease of the sum of the values of model selection criterion and searches the model minimizing (19), although it cannot guarantee the decreasing of the loss function.

## Results and Discussion

We tested the performance of the proposed method on both simulated and real gene expression time-course data. Using simulated data, we showed the differences between the proposed method and other *l*_1_-regularization approaches in the variable selection problem for linear regression model and evaluated how well the proposed method estimates the coefficient matrix of the VAR model compared with other estimation methods. Using real data, we also compared the proposed method with various reverse-engineering algorithms for inferring gene networks.

### Simulation Results

#### Simulation 1

We compared our proposed methods, the recursive elastic net (REN) and the corrected recursive elastic net (CREN), with the other *l*_1_-regularization methods, the lasso (LA), the naive elastic net (NEN) and the elastic net (EN) on simulated data in linear regression. We generated data from a linear regression model

(20)

where β* was a 1000-dimensional vector of coefficients, and ***x ***followed a 1000-dimensional multivariate normal distribution *N*(**0**, ***S***) with the zero mean vector **0 **and the covariance matrix ***S ***whose (*i*, *j*)-th entry was *S*_*i*, *j *_= 0.3 if *i *≠ *j *and *S*_*i*, *i *_= 1 otherwise. The true coefficient vector was , *i.e.*, the first five coefficients were related with the response *z*. The first five entries  were generated from a uniform distribution on the interval [-1.2, -0.8] and [0.8, 1.2]. The standard deviation *σ *was chosen so that the signal-to-noise ratio was 10, and 50 observations were generated from this model. We set the grid of the values of the *l*_2_-regularization parameters to {0.5, 1, 2, 5}. The regularization parameters of each method were selected by a model selection criterion AICc. We used AICc as a stopping criterion of REN and CREN and the algorithm stopped if AICc was not decreasing in the next iteration. All the methods were run on each dataset through 100 simulations. Results of Simulation 1 are described in Table 1. TDR stands for true discovery rate (or accuracy) defined as TDR = TP/(TP + FP) and sensitivity (SE) is TP/(TP+FN), where TP, FP, TN and FN are the number of true positives, false positives, true negatives and false negatives, respectively. Compared with REN and CREN with the other *l*_1_-regularization methods, LA, NEN and EN had larger numbers of true positives than REN and CREN. However, LA, NEN and EN also had larger numbers of false positives, and they suffered from their low true discovery rates. While REN and CREN had the higher true discovery rates than LA, NEN and EN, since they reduced the numbers of false positives drastically while keeping the numbers of true positives as large as possible. Especially, REN reduced the number of false positives from 11.90 to 1.01 and had the highest true discovery rate between all the algorithms.

**Table 1:** Results of Simulation 1

Method	TP	FP	TN	FN	TDR	SE
LA	**4.60**	25.38	969.62	**0.40**	0.22	**0.92**
NEN	4.38	11.90	983.10	0.62	0.30	0.88
EN	4.22	7.89	987.11	0.78	0.40	0.84
REN	4.07	**1.01**	**993.99**	0.93	**0.84**	0.81
CREN	3.78	1.05	993.95	1.22	**0.84**	0.76

**Figure 1:**
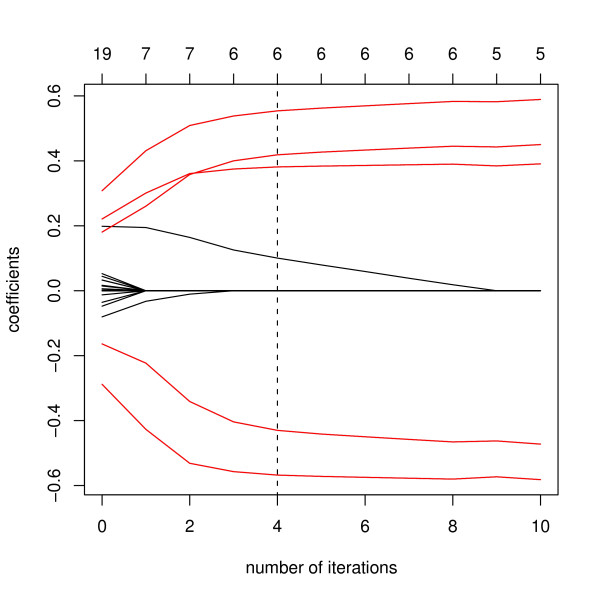
**Coefficient profiles of the recursive elastic net through 10 iterations**. The numbers on the top of the figures are the numbers of nonzero coefficients at each iteration. The red lines indicate variables whose coefficients are nonzero in the true model. The black lines are noisy variables. The dot line shows the iteration when the stopping criterion is fulfilled.

Next we illustrates the difference between the naive elastic net and the recursive elastic net on a dataset from the model (20). Figure 1 illustrates the coefficient profiles through 10 iterations with the application of the recursive elastic net on a dataset of 50 observations. The red lines indicate the first 5 coefficient profiles related with the response *z*, that is, the five true coefficient profiles. While the black lines indicate the noisy coefficient profiles. The dot line stands for the iteration when the stopping criterion was fulfilled.

The coefficients of the 0-th iteration was equivalent to the naive elastic net estimators where 19 coefficients were identified as nonzero including the 5 true components (true positives) and 14 noisy components (false positives). In the first iteration, among the 19 components selected in the naive elastic net, 7 coefficients including the 5 true components were estimated as nonzero by the recursive elastic net. That is, we succeeded in reducing the number of false positives from 14 to 4 while keeping all the 5 true positives. After 5 iterations, the stopping criterion was fulfilled and the recursive elastic net yielded 5 true positives and only 1 false positive. This reduction of the number of false positives in the linear regression has much effect on the performance of the VAR model which is equivalent to *m *linear regressions. The performances of the proposed methods in the VAR model will be illustrated in the next section.

#### Simulation 2

For the second simulation, we used the VAR model with the structure of scale-free networks. We considered five models with (*n*, *m*) = (20, 100), (20, 200), (20, 500), (20, 1000) and (20, 2000) where *n *was the number of time points and *m *was the number of variables. The topologies of the scale-free networks were generated according to the Barabasi-Albert model [26] by using the R function barabasi.game in the R package igraph [27]. The parameter power was set to 1.2 and the other parameters were set to their default values, and then the numbers of edges were chosen to be 108, 213, 525, 1045 and 2079 for *m *= 100, 200, 500, 1000 and 2000, respectively. The structures of the simulated networks are illustrated in Additional File 1. The dataset from the VAR model with a given structure was drawn as follows:

1. The nonzero entities for the true VAR coefficients B* were generated from a uniform distribution on the interval [-0.8, -0.4] and [0.4, 0.8].

2. The initial value ***y***_0 _was drawn from a uniform distribution on the interval [10, 100].

3. The data at time points *t *∈ {1, 2, ..., 20} was generated recursively according to the VAR model with identity random noise matrix **Σ **= ***I***.

We simulated 100 different datasets from the above procedure for each combination of (*n*, *m*). We compared the recursive elastic net (REN) and the corrected recursive elastic net (CREN) with the lasso (LA), the naive elastic net (NEN), the elastic net (EN), and the Jame-Stein shrinkage (JS) [7] on simulated datasets. Opgen-Rhein and Strimmer [7] proposed an improved estimator of the VAR coefficients by using a James-Stein-type shrinkage approach. All the algorithms were run out on each drawn dataset {***y***_1_, ..., ***y***_20_}. The setting of the *l*_2_-regularization parameters was same as given in Simulation 1. The regularization parameters of LA, NEN, EN, REN and CREN were selected by a model selection criterion, BIC or AICc. JS is a test-based approach and a cut-off value for the local false discovery was set to 0.2 (JS-A), which was used in Opgen-Rhein and Strimmer [7]. We also used a cut-off value such that the number of significant edges detected by JS was as same as that of REN with AICc (JS-B).

For each algorithm, its network inference performance was evaluated by true discovery rate (TDR = TP/(TP + FP) and sensitivity (SE = TP/(TP + FN)) where TP, FP, TN and FN are the number of true positives, false positives, true negatives and false negatives, respectively. Table 2 presents the results of Simulation 2. These results can be summarized as follows:

**Table 2:** Results of Simulation 2

		number of time points × number of variables									
		
		20 × 100		20 × 200		20 × 500		20 × 1000		20 × 2000	
						
Method	MC	TDR	SE	TDR	SE	TDR	SE	TDR	SE	TDR	SE
LA	BIC	0.06	0.77	0.05	0.72	0.05	0.69	0.05	0.67	0.05	0.65
LA	AICc	0.08	0.78	0.07	0.76	0.07	0.73	0.06	0.71	0.06	0.69
NEN	BIC	0.08	**0.93**	0.07	**0.94**	0.06	**0.91**	0.06	**0.88**	0.06	**0.84**
NEN	AICc	0.12	**0.93**	0.10	0.93	0.08	**0.91**	0.08	0.87	0.07	0.83
EN	BIC	0.25	0.91	0.19	0.91	0.15	0.89	0.12	0.85	0.11	0.82
EN	AICc	0.25	0.91	0.20	0.91	0.15	0.89	0.13	0.85	0.11	0.82
REN	BIC	0.74	0.73	0.71	0.70	0.68	0.68	0.65	0.65	0.61	0.61
REN	AICc	**0.77**	0.75	**0.73**	0.72	**0.70**	0.69	**0.67**	0.66	**0.63**	0.62
CREN	BIC	0.54	0.82	0.44	0.81	0.33	0.81	0.27	0.78	0.22	0.76
CREN	AICc	0.56	0.82	0.45	0.81	0.34	0.81	0.28	0.78	0.23	0.76
JS-A	-	0.09	0.79	0.05	0.74	0.02	0.75	0.01	0.74	4.7 × 10^-2^	0.74
JS-B	-	0.29	0.29	0.19	0.19	0.11	0.10	0.05	0.05	0.03	0.03

1. The sensitivity and true discovery rate of each algorithm were gradually decreasing as increasing the number of variables.

2. NEN and EN had higher sensitivities than the other methods, but their true discovery rates were much lower than those of REN and CREN.

3. The sensitivity of LA was slightly higher than that of REN, while the true discovery rate of REN was over ten times higher than that of LA.

4. Compared with the same significant edges of REN and JS-B, REN outperformed JS-B in both of sensitivity and true discovery rate.

5. CREN succeeded in higher sensitivity than REN, but it suffered from its low true discovery rate compared with REN when the number of variables was large.

6. REN had the highest true discovery rate between all the algorithms in all cases while its sensitivity was almost same as that of LA, JS-A and CREN.

7. REN with AICc was slightly better than REN with BIC.

We also compared our proposed method with other competing methods on simulated dataset by using the VAR model with hierarchical structures. These results are given in Additional File 2.

### Experimental Results

In this section we used experimental data of MCF-7 breast cancer cells stimulated with two ErbB ligands, epidermal growth factor (EGF) and heregulin (HRG), to compare the proposed method with various reverse-engineering algorithms. This dataset is available from Gene Expression Omnibus (GEO, Accession Number GSE6462) [28]. In GSE6462, cells were stimulated with 0.1, 0.5, 1, or 10 nM of either EGF or HRG and the expression values were measured at eight time points (0, 5, 10, 15, 30, 45, 60, and 90 minutes) by Affymetrix GeneChip U133A-2. The expressions with 10 nM of EGF at 60 minutes were not observed, and thus its values were estimated with linear interpolation. These microarray datasets were also normalized by faster cyclic loess [29].

In addition to the lasso, the naive elastic net, the elastic net, and the James-Stein shrinkage which were used in Simulation Section, we considered MRNET [30], CLR [2], and ARACNE [31] as competing methods. These methods are available from the R package minet [32]. Note that they do not intend to analyze time-course data. To increase their capabilities, we considered a lagged version of a time series matrix, denoted by ***D***, whose (*i*, *j*)-element indicates *D*_*i*, *j *_= *y*_*i*+1, *j *_- *y*_*i*, *j *_and used it as an input of MRNET, CLR and ARACNE. This modification enables us to extract gene-gene interactions between time steps *i *+ 1 and *i*. To distinguish between the original version and the modified version, we refer the original MRNET, CLR and ARACNE as MRN, CLR and ARA, and the modified MRNET, CLR and ARACNE as MRN-L, CLR-L and ARA-L, respectively. In our analysis, we assumed that time points were equidistant and that the structure of gene networks did not change with different dosages of EGF or HRG. Thus, the datasets from different dose conditions of EGF or HRG were considered as replicated observations. We validated the performances of these reverse-engineering algorithms in terms of how to identify pairwise gene-gene interaction edges by using TRANSPATH database [33]. Although we do not have perfect knowledge of true network, we used biological interactions obtained from TRANSPATH database as true edges. We started by focusing on 254 probes by Nagashima *et al. *[34]. Among these probes, 233 probes were mapped to unique Entrez Gene IDs, 7 probes were mapped to more than one Entrez Gene IDs, and 14 probes were not mapped, respectively. We only used 233 probes with 228 unique Entrez Gene IDs. To correspond each Entrez Gene ID to unique probe, we selected a probe for each Entrez Gene ID with the largest mean value of expressions through all the experiments. Among these 228 Entrez Gene IDs, TRANSPATH database identified a biological pathway which includes 110 Entrez Gene IDs. While the other 128 Entrez Gene IDs do not connect each other in TRANSPATH database. Thus, all the algorithms were run on time-course expression profiles of the 110 Entrez Gene IDs for EGF-stimulation and HRG-stimulation, respectively.

The setting of the regularization parameters in the lasso (LA), the naive elastic net (NEN), the elastic net (EN), the recursive elastic net (REN) and the corrected recursive elastic net (CREN) was same as given in Simulation and these parameters were selected by BIC. As a result, LA, NEN, EN, REN and CREN identified 2202, 1721, 870, 162 and 436 significant edges from the EGF dataset, respectively. While they also identified 2904, 2335, 411, 154 and 171 significant edges from the HRG dataset, respectively. In order to facilitate comparison among them, we controlled the numbers of significant edges detected by LA, NEN, EN and CREN same as that of REN by using a threshold value, that is, set to 0 all edges whose coefficients are lower than the threshold. We also chose the same number of significant edges detected by the James-Stein shrinkage (JS) as that of REN in a similar way as in the previous section. MRN, CLR, ARA, MRN-L, CLR-L and ARA-L require the calculation of a mutual information matrix. We used a Miller-Madow asymptotic bias corrected empirical estimator as each mutual information of them. We set the numbers of significant edges detected by them as same as that of REN by using a threshold. Notice that each of MRN, CLR, ARA, MRN-L, CLR-L and ARA-L infers the network just as an undirected graph, while each of the others infers the network as a directed graph. In our analysis, we first transformed the real and the inferred directed networks into undirected graphs and then performed a comparison. Results from experimental data are described in Table 3. TDR stands for true discovery rate defined as TP = (TP + FP) where TP and FP are the numbers of true positives and false positives, respectively. The method RAND is an algorithm which selects the same number of significant edges same as that of REN randomly. Each value of RAND indicates the mean value through 1,000,000 simulations. The *p*-value of each algorithm was calculated using a Binomial distribution based on the random model through 1,000,000 simulations. Notice that the overall low performances on the EGF and HRG datasets are owing to the limited number of data and the imperfect knowledge of the real network. We observed that, in the EGF dataset, ARA, MRN-L, LA, NEN, REN and CREN had higher numbers of true positives than RAND, however only the performance of REN was significantly better than that of RAND. While, in the HRG dataset, MRN-L, CLR-L, ARA-L, NEN, EN, REN and CREN performed well than RAND. Between them, the performances of EN, REN and CREN were significantly better than that of RAND. As a result, REN had the highest number of true positives on both of the EGF and HRG datasets.

**Table 3:** Results of the applications of network inference algorithms on the experimental datasets

Method	EGF			HRG		
		
	TP	TDR	*p*-value	TP	TDR	*p*-value
RAND	8.73	0.05	-	8.29	0.05	
MRN	8	0.05	0.65	8	0.05	0.59
CLR	4	0.03	0.98	7	0.05	0.73
ARA	9	0.06	0.51	7	0.05	0.73
MRN-L	12	0.08	0.16	10	0.07	0.32
CLR-L	6	0.04	0.87	9	0.06	0.45
ARA-L	6	0.04	0.87	9	0.06	0.45
JS	6	0.04	0.87	8	0.05	0.59
LA	9	0.06	0.51	7	0.05	0.73
NEN	10	0.06	0.38	9	0.06	0.45
EN	8	0.05	0.65	**17**	**0.11**	**4.0 **× **10^-3^**
REN	**16**	**0.10**	**0.01**	**19**	**0.13**	**6.7 **× **10^-4^**
CREN	11	0.07	0.25	**16**	**0.11**	**8.8 **× **10^-3^**

Next we applied our method to time-course expression profiles of the 228 Entrez Gene IDs stimulated with EGF and HRG, respectively, in order to investigate the difference between the EGF- and HRG-induced gene networks. In the ErbB signaling network, two different ErbB receptor ligands, EGF and HRG, play a key role in controlling diverse cell fates. In MCF-7 cells, stimulation with EGF causes sustained network activation and leads to cell differentiation, while stimulation with HRG causes transient network activation and leads to cell proliferation.

**Figure 2:**
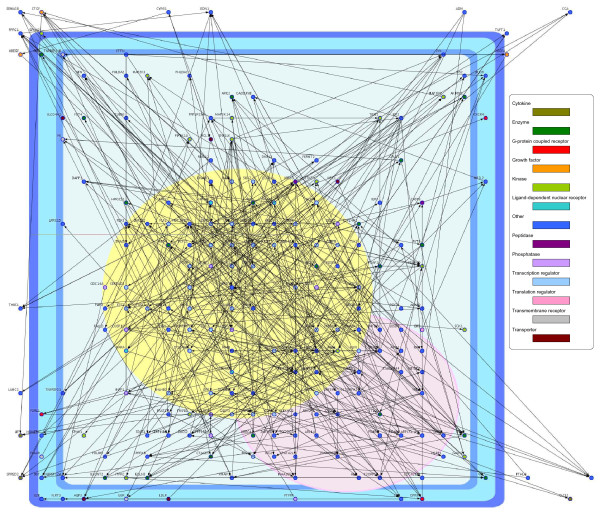
**EGF-induced VAR network inferred from time-course microarray data in MCF-7 cells**. The nodes indicate genes and the edges represent functional connectivities.

**Figure 3:**
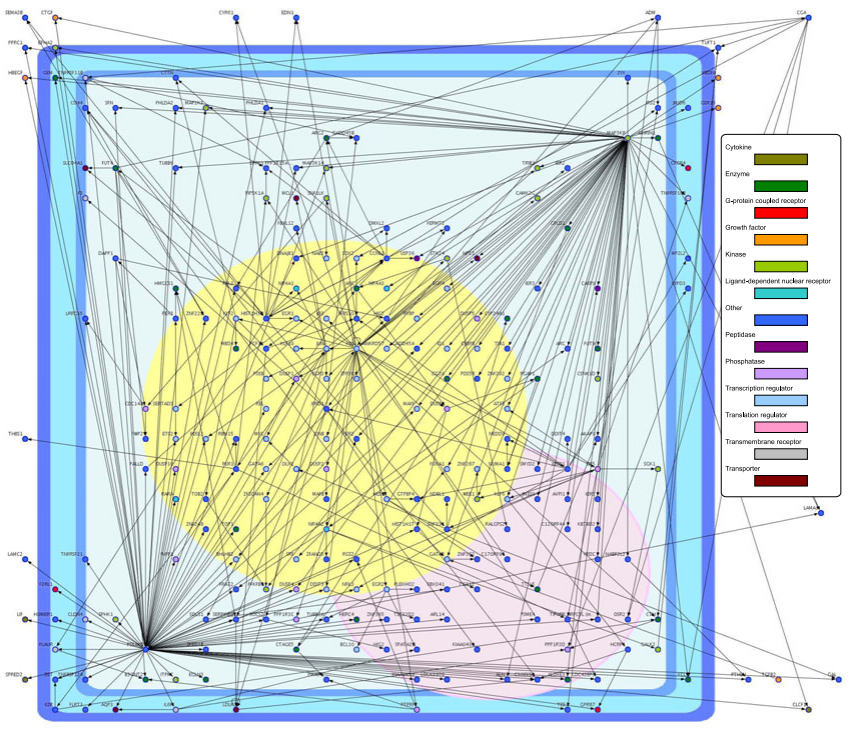
**HRG-induced VAR network inferred from time-course microarray data in MCF-7 cells**. The nodes indicate genes and the edges represent functional connectivities.

Figures 2 and 3 show the inferred EGF- and HRG-induced gene networks with the recursive elastic net.

These figures were illustrated by Cell Illustrator [35,36]. We observed that the two directed graphs had different network topologies each other. We compared with the two graphs from characteristics of node degree, that is, the number of edges that they had. In directed networks, we distinguish between the in-degree, the number of directed edges that point toward the node, and the out-degree, the number of directed edges that start at the node. The in-degree distributions and the out-degree distributions of the EGF- and HRG-induced networks are illustrated in Additional File 3. We found that each of the in-degree distributions concentrated around its average value, whereas the out-degree distributions had long tails. In particular, the out-degree distribution of the HRG-induced network was more heavy tailed than that of the EGF-induced network. Furthermore, in the HRG-induced network, we found that three hub genes, *FOS*, *MAP3K8 *and *PDLIM5*, had larger out-degrees (30, 68 and 75, respectively) than the other genes. While, in the EGF-induced network, each of *FOS*, *MAP3K8 *and *PDLIM5 *had only one outgoing edge (one out-degree). Thus, these hub genes are thought to be essential in only the HRG-induced network since their disruptions do not lead to a major loss of connectivity in the EGF-induced network, but the loss of them causes the breakdown of the HRG-induced network into isolated clusters. Our analysis based on the VAR model with the proposed method produced the hypothesis that these hub genes might be implicated why only HRG allows MCF-7 cells to be differentiated.

## Conclusion

In this article, we have proposed a new class of *l*_1_-regularization, called recursive elastic net for inferring gene networks from time-course microarray data based on the VAR model. The recursive elastic net addresses the drawback of the elastic net and has higher true discovery rate than other competing methods in gene network inference. Numerical simulations demonstrated that the proposed method succeeded in reducing the number of false positives drastically while keeping the large number of true positives and achieved two or more times higher true discovery rate than the lasso, the naive elastic net, the elastic net and the James-Stein shrinkage even when the number of time points was small. We also compared our method with various reverse-engineering algorithms including MRNET, CLR and ARACNE by using experimental data of MCF-7 breast cancer cells and TRANSPATH database. As a result, we found that the proposed method had the best performance between them and provided some differences between the EGF- and HRG-induced networks.

An interesting direction for future work would be to incorporate prior information elicited from the known biological networks. In the recursive elastic net, we can incorporate the topology of the known network directly as the initial variable weights (10). For example, if an edge from gene *i *to gene *j *exists the known biological pathway, we impose a small weight less than one on the corresponding coefficient. This incorporation would further provide higher sensitivity and higher true discovery rate of the proposed method in gene network inference. Another promising direction for future work would be the extension of the recursive elastic net to other type of mathematical models. A limitation of the VAR model comes from their equidistant assumption which is not true in many real situations. We might solve the problem by applying the proposed method to differential equation models [37,38].

## Appendix: Proof of Theorem 1

We provide proof of Theorem 1 which is similar to that of Zou and Hastie [13]. If , then both  and  are nonzero, and we have . Because of (10), the estimator  satisfies



Hence we have

(21)

(22)

Subtracting (21) from (22) gives



which is equivalent to

(23)

Using the identity |*A *± *B*| ≤ |*A*| + |*B*| for all *A *and *B*, we have



By equation (6) we must have



that is,



So



Then equation (23) implies

(24)

If we assume that ***X ***is standardized,  where . Thus inequality (24) reduces to

(25)

We assume that . Since the function *f*(*x*) = *x*^-1 ^is Lipshitz continuous for *x *> 0, then we have

(26)

Using (26) gives



Thus inequality (25) further simplifies to

(27)

This completes the proof.

## Authors' contributions

TS conceived and designed the study, and wrote the manuscript. SI provided statistical expertise and careful manuscript review. RY, AF and MN assisted in preparing the manuscript. SM supervised the whole project. All authors read and approved of the final manuscript.

## Supplementary Material

Additional file 1 **Structures of simulated scale-free networks**. This file includes Additional Figures 1, 2, 3, 4 and 5 that describe the structures of the simulated scale-free networks with 100, 200, 500, 1000 and 2000 genes, respectively. Click here for file

Additional file 2 **Simulation 3**. This document represents Simulation 3 where we compared our proposed method with other competing methods on simulated dataset by using the VAR model with a hierarchical structure. It also includes Additional Figure 6 that describes the structure of the simulated network and Additional Tables 1 and 2 that show the results of Simulation 3, respectively. Click here for file

Additional file 3 **In-degree and out-degree distributions of the EGF- and HRG-induced gene networks**. This file includes Additional Figures 7 and 8 that describe the in-degree distribution and the out-degree distribution of the EGF-induced gene network, and Additional Figures 9 and 10 that describe the in-degree distribution and the out-degree distribution of the HRG-induced gene network, respectively. Click here for file
