# Supplementary material for: Recursive regularization for inferring gene networks from time-course gene expression profiles
Source: BMC Syst Biol. 2009 Apr 22;3:41. doi: 10.1186/1752-0509-3-41 (PMC2686685; doi:10.1186/1752-0509-3-41)

Additional File 1: Structures of Simulated Scale-Free Networks

Additional Figure 1 – An example of the simulated network for the setting of $m = 100$

The solid lines indicate positive coefficients and the dotted lines negative coefficients in the coefficient matrix of the VAR model.

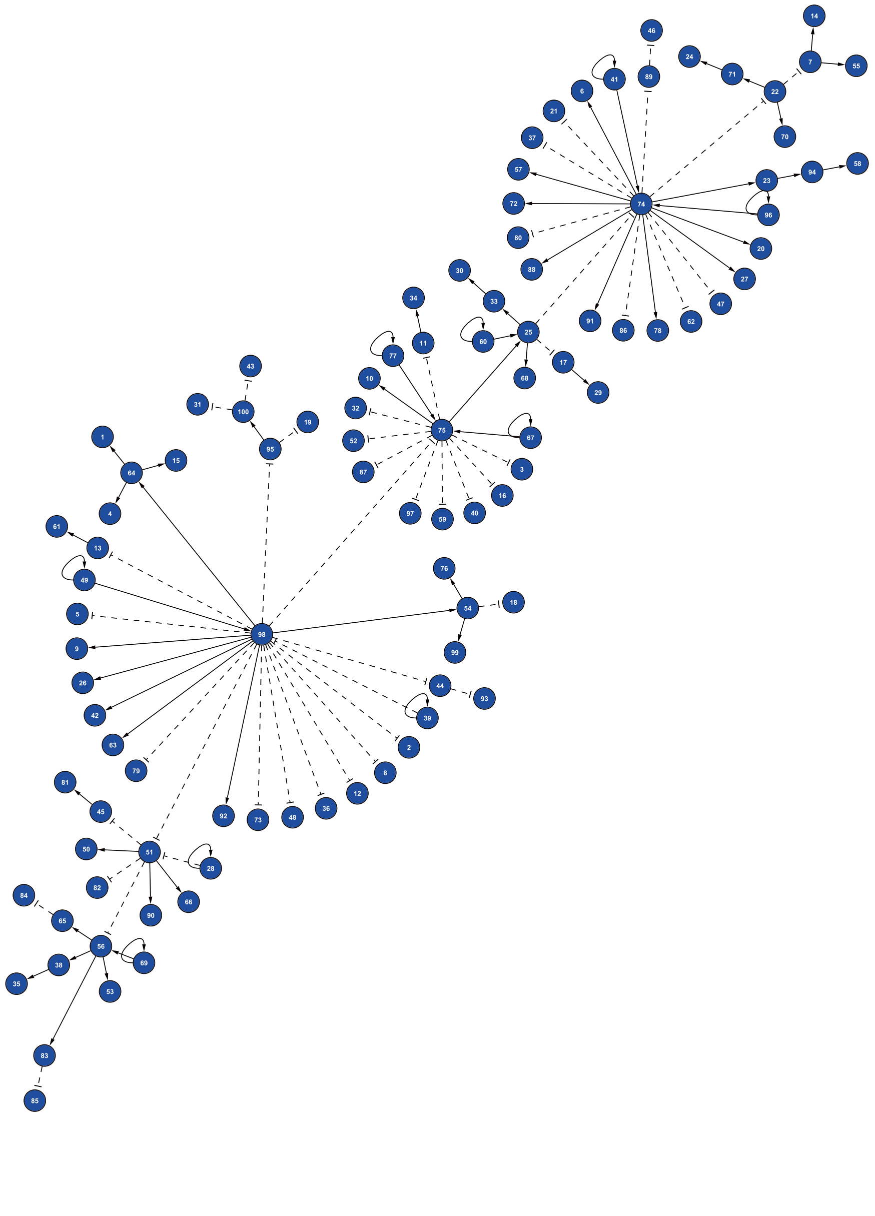

Additional Figure 2 – An example of the simulated network for the setting of $m = 200$

The solid lines indicate positive coefficients and the dotted lines negative coefficients in the coefficient matrix of the VAR model.

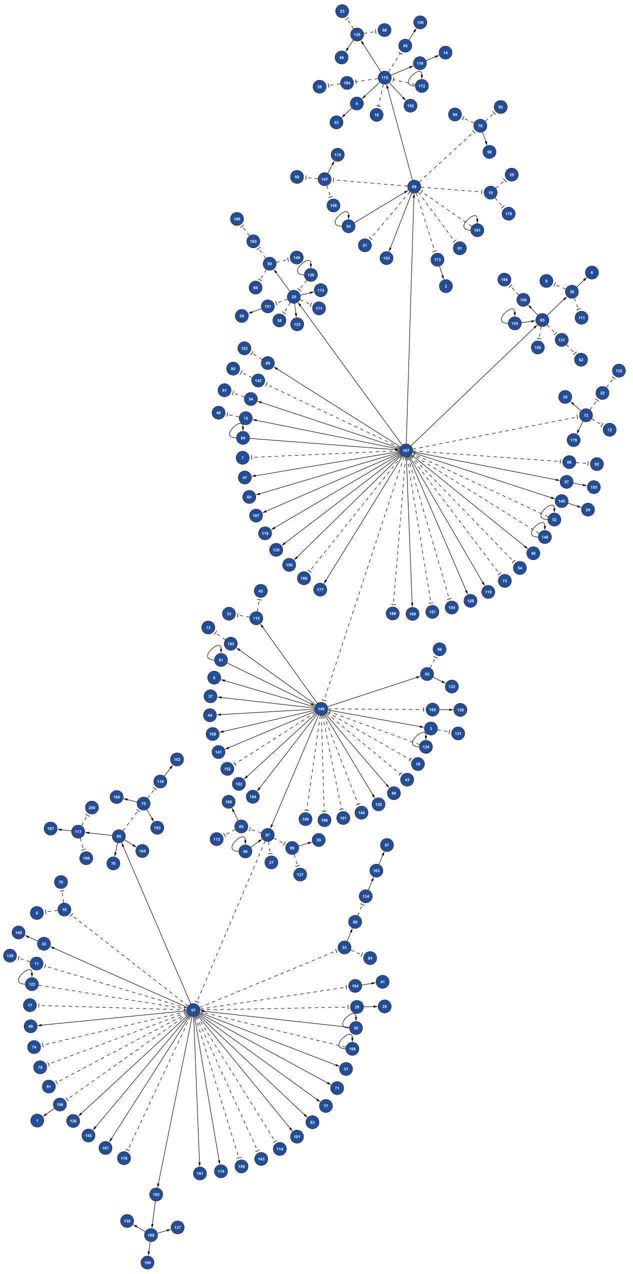

Additional Figure 3 – An example of the simulated network for the setting of $m = 500$

The solid lines indicate positive coefficients and the dotted lines negative coefficients in the coefficient matrix of the VAR model.

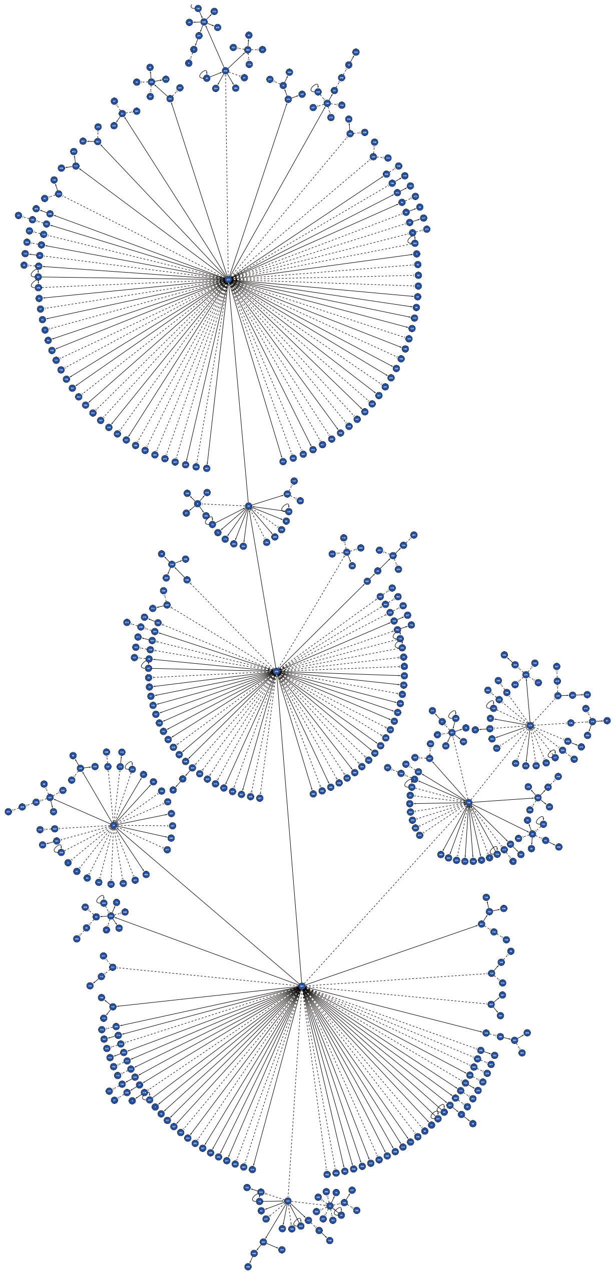

Additional Figure 4 – An example of the simulated network for the setting of $m = 1000$

The solid lines indicate positive coefficients and the dotted lines negative coefficients in the coefficient matrix of the VAR model.

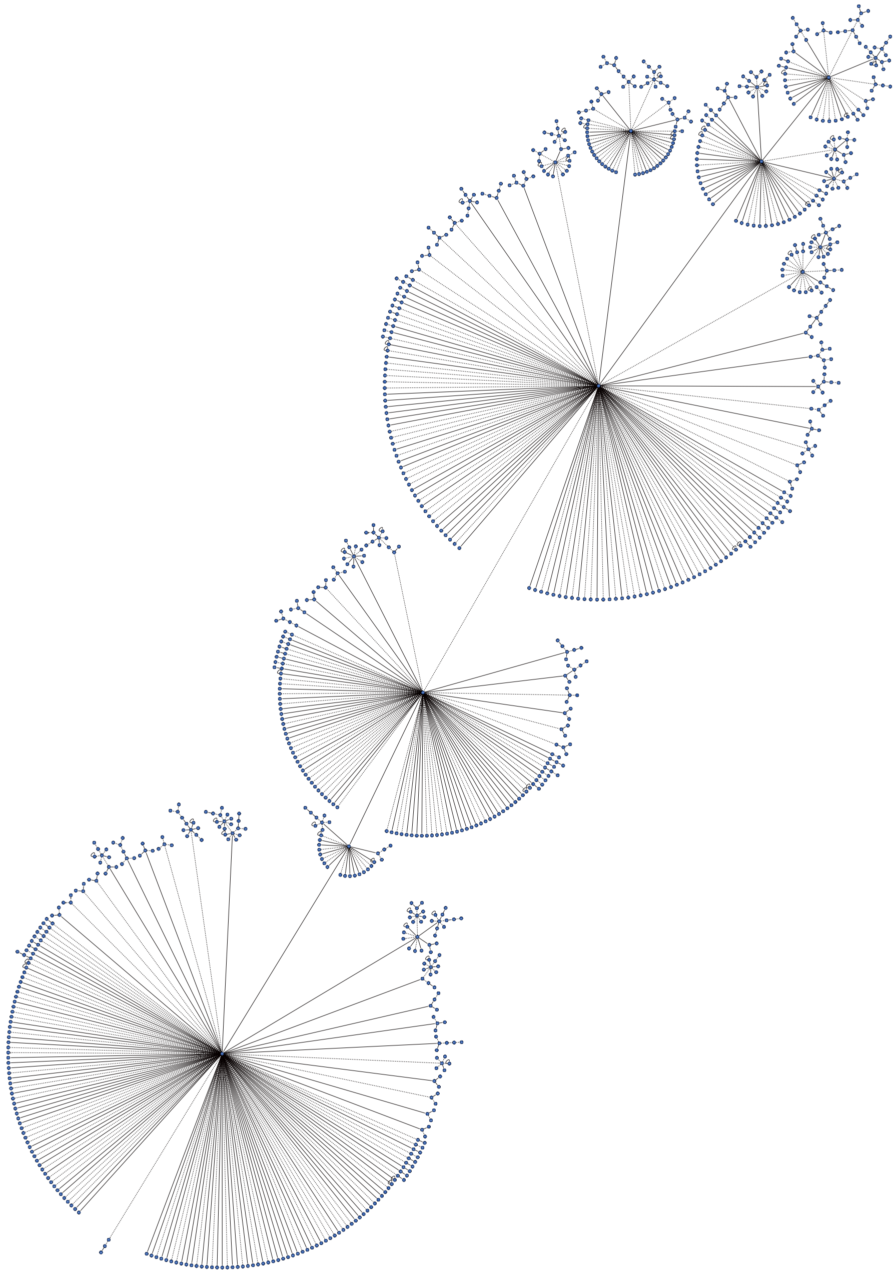

Additional Figure 5 – An example of the simulated network for the setting of $m = 2000$

The solid lines indicate positive coefficients and the dotted lines negative coefficients in the coefficient matrix of the VAR model.

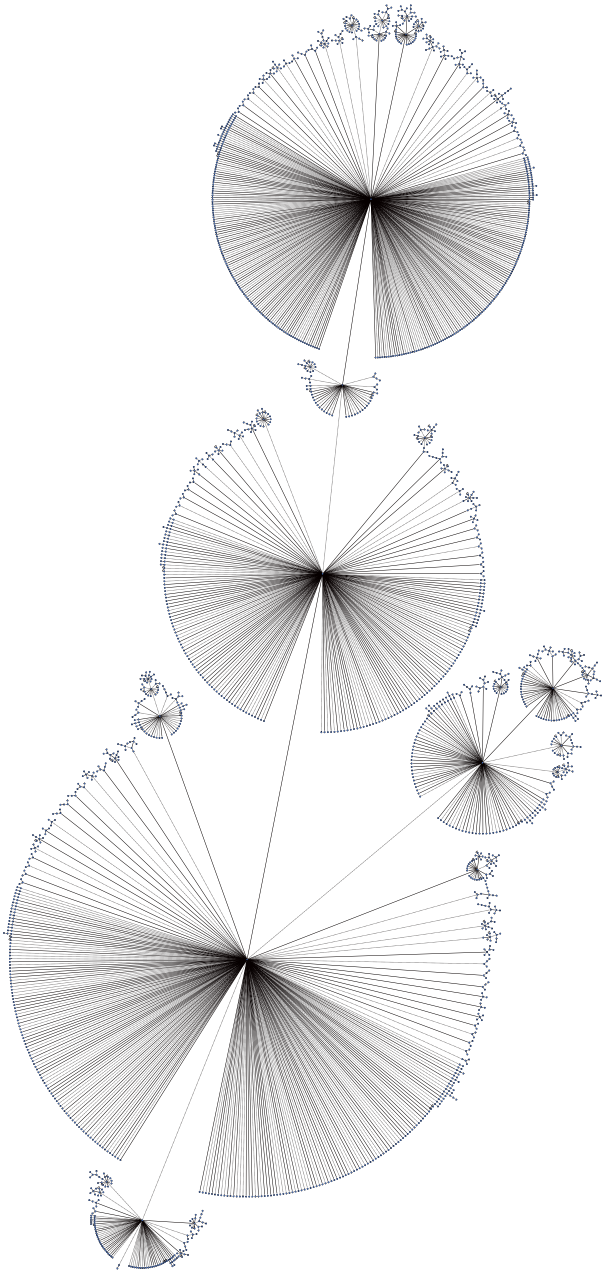

Supplement: Additional file 1 — Structures of simulated scale-free networks. This file includes Additional Figures 1, 2, 3, 4 and 5 that describe the structures of the simulated scale-free networks with 100, 200, 500, 1000 and 2000 genes, respectively. [file 1752-0509-3-41-S1.pdf]
