# Supplementary material for: Recursive regularization for inferring gene networks from time-course gene expression profiles
Source: BMC Syst Biol. 2009 Apr 22;3:41. doi: 10.1186/1752-0509-3-41 (PMC2686685; doi:10.1186/1752-0509-3-41)
Supplement: Additional file 2 — Simulation 3. This document represents Simulation 3 where we compared our proposed method with other competing methods on simulated dataset by using the VAR model with a hierarchical structure. It also includes Additional Figure 6 that describes the structure of the simulated network and Additional Tables 1 and 2 that show the results of Simulation 3, respectively. [file 1752-0509-3-41-S2.pdf]

Additional File 2: Simulation 3

We compared our methods, the recursive elastic net (REN) and the corrected recursive elastic net (CREN), with other competing approaches, the lasso (LA), the naive elastic net (NEN), the elastic net (EN) and the James-Stein shrinkage (JS) on simulated data from the VAR model with hierarchical structures illustrated in Additional Figure 6.

The mathematical equation of the hierarchical VAR model is given as follows:

$$\begin{aligned}\boldsymbol{y}_t &= \boldsymbol{B}'\boldsymbol{y}_{t-1} + \boldsymbol{\epsilon}_t,\ \boldsymbol{\epsilon}_t \sim N(\mathbf{0}, 0.4\boldsymbol{I}) \\ \boldsymbol{B} &= \begin{pmatrix} \boldsymbol{B}^{(1)} & \boldsymbol{B}^{(2)} & \mathbf{0} \\ \mathbf{0} & \boldsymbol{B}^{(3)} & \mathbf{0} \\ \mathbf{0} & \mathbf{0} & \mathbf{0} \end{pmatrix}\end{aligned}$$

where

$$\boldsymbol{B}^{(1)} = \begin{pmatrix} 1 & 0 & 0 & 0 & 0 \\ 0 & 0.9 & 0.2 & 0 & 0 \\ 0 & 0 & 0.4 & -0.1 & 0 \\ 0 & 0 & 0 & 0.95 & 0 \\ 0.5 & 0 & 0 & 0.9 & 0 \end{pmatrix}$$

$$\boldsymbol{B}^{(2)} = \begin{pmatrix} \overbrace{1,\dots,1}^{10} & \mathbf{0} & \mathbf{0} & \mathbf{0} & \mathbf{0} \\ \mathbf{0} & \overbrace{1,\dots,1}^{10} & \mathbf{0} & \mathbf{0} & \mathbf{0} \\ \mathbf{0} & \mathbf{0} & \overbrace{1,\dots,1}^{10} & \mathbf{0} & \mathbf{0} \\ \mathbf{0} & \mathbf{0} & \mathbf{0} & \overbrace{1,\dots,1}^{10} & \mathbf{0} \\ \mathbf{0} & \mathbf{0} & \mathbf{0} & \mathbf{0} & \overbrace{1,\dots,1}^{10} \end{pmatrix}$$

$$\boldsymbol{B}^{(3)} = \begin{pmatrix} \overbrace{1,\dots,1}^{10} & \mathbf{0} & \mathbf{0} & \cdots & \mathbf{0} \\ \overbrace{0,\dots,0}^{5}\overbrace{1,\dots,1}^{5} & \overbrace{1,\dots,1}^{5}\overbrace{0,\dots,0}^{5} & \mathbf{0} & \cdots & \mathbf{0} \\ \mathbf{0} & \overbrace{1,\dots,1}^{10} & \mathbf{0} & \cdots & \mathbf{0} \\ \mathbf{0} & \overbrace{0,\dots,0}^{5}\overbrace{1,\dots,1}^{5} & \overbrace{1,\dots,1}^{5}\overbrace{0,\dots,0}^{5} & \cdots & \mathbf{0} \\ \vdots & \vdots & \vdots & \ddots & \vdots \\ \mathbf{0} & \mathbf{0} & \mathbf{0} & \cdots & \overbrace{1,\dots,1}^{10} \end{pmatrix}$$

The number of variables was 330 and 50 time points were generated from this model. The simulated model has three-level structures and the variables of the third layer form several groups whose underlying factors are the variables of the first layer. For example, the variables 61, 62, 63, 64 and 65 are considered as a group of the variable 1, and we want to identify the variables 6 and 7 whose parent is the variable 1. The

dataset was generated in the same procedure as in Simulation 2. The setting of the regularization parameters was also as same as that of Simulation 2.

The result of Simulation 3 is presented in Additional Table 1. We observed that LA and JS-A detected the larger numbers of false positives than the other methods and thus had the poor performances of true discovery rates. EN improved the performance of NEN in terms of the number of false positives and true discovery rate, while the performance of CREN was poor than that of REN. In Simulation 3, the scale factor of CREN looks like its not working well. In a similar fashion to Simulation 2, REN achieved the extra higher sensitivity and true discovery rate simultaneously.

We further investigated the performances of other variable weights for REN on the simulated datasets in the setting of Simulation 3. We considered the four other weights. The weight rand stands for random weights generated from a uniform distribution on the interval $[0, 1]$. The weight ridge, lasso, and enet correspond to the inverses of the ridge, the lasso and the elastic net estimators as the initial weights, respectively. The weight nenet is equivalent to the initial weights for the original recursive elastic net.

The results of the performances with different initial variable weights are given in Additional Table 2. We observed that the performance using the random weights was poor compared with those of the other weights. This implies that an unsuitable starting point leads to a poor estimator in the recursive elastic net. While the weights with the lasso, the naive elastic net and the elastic net reached high sensitivities and high true discovery rates. They also outperformed the ridge estimator. In particular, the naive elastic net had the best performance between them. As a result, we found that our proposed variable weights using the naive elastic net make sense as a initial value of variable weights to some extent.

Additional Figure 6 – An example of the simulated network for the setting of $m = 330$

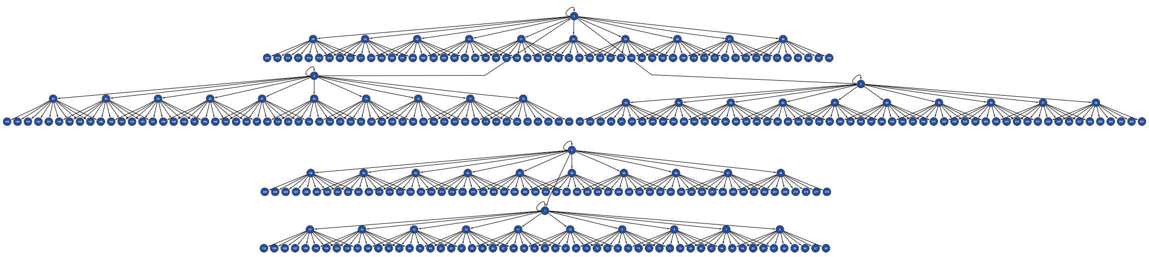

Additional Table 1 - Results of Simulation 3

Method	MC	TP	FP	TN	FN	TDR	SE
LA	BIC	555.40	10945.10	97396.90	2.60	0.05	**1.00**
LA	AICc	525.16	8991.56	99350.44	32.84	0.06	0.94
NEN	BIC	555.84	3679.64	104662.36	2.16	0.14	**1.00**
NEN	AICc	**556.00**	3377.88	104964.12	**2.00**	0.15	**1.00**
EN	BIC	555.13	940.35	107401.65	2.87	0.42	0.99
EN	AICc	555.17	950.87	107391.13	2.83	0.42	0.99
REN	BIC	546.49	**34.89**	**108307.11**	11.51	**0.94**	0.98
REN	AICc	549.44	58.24	108283.76	8.56	0.91	0.98
CREN	BIC	392.63	111.73	108230.27	165.37	0.85	0.70
CREN	AICc	393.94	113.07	108228.93	164.06	0.85	0.71
JS-A	–	468.54	8532.94	99809.06	89.46	0.06	0.84
JS-B	–	346.07	235.31	108106.69	211.93	0.60	0.62

Additional Table 2 - Results of Comparison with Different Variable Weights

Method	MC	Weight	TP	FP	TN	FN	TDR	SE
REN	AICc	rand	392.48	13054.90	95287.10	165.52	0.03	0.70
REN	AICc	ridge	444.73	109.73	108232.30	113.27	0.81	0.80
REN	AICc	lasso	**553.61**	62.59	108279.40	**4.39**	0.90	**0.99**
REN	AICc	nenet	549.44	58.24	108283.76	8.56	0.91	0.98
REN	AICc	enet	542.24	85.38	108256.60	15.76	0.87	0.97
REN	BIC	rand	365.93	12704.99	95637.01	192.07	0.03	0.66
REN	BIC	ridge	441.93	70.29	108271.71	116.07	0.87	0.79
REN	BIC	lasso	548.03	46.24	108295.76	9.97	0.92	0.98
REN	BIC	nenet	546.49	**34.89**	**108307.11**	11.51	**0.94**	0.98
REN	BIC	enet	539.46	55.54	108286.50	18.54	0.91	0.97
