# Supplementary material for: Recursive regularization for inferring gene networks from time-course gene expression profiles
Source: BMC Syst Biol. 2009 Apr 22;3:41. doi: 10.1186/1752-0509-3-41 (PMC2686685; doi:10.1186/1752-0509-3-41)

Additional File 3: In-Degree and Out-Degree Distributions of the EGF- and HRG-induced Gene Networks

Additional Figure 7 – In-Degree Distribution of the EGF-Induced Gene Network

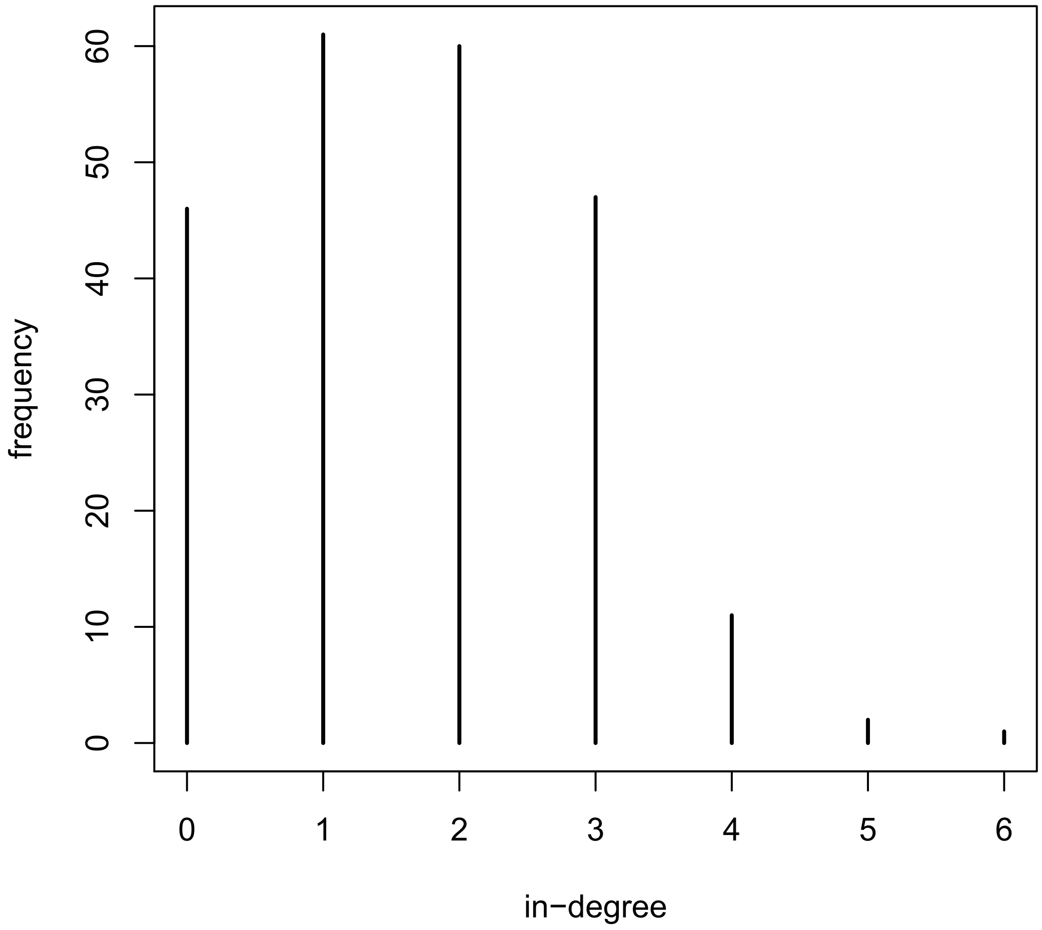

Additional Figure 8 – Out-Degree Distribution of the EGF-Induced Gene Network

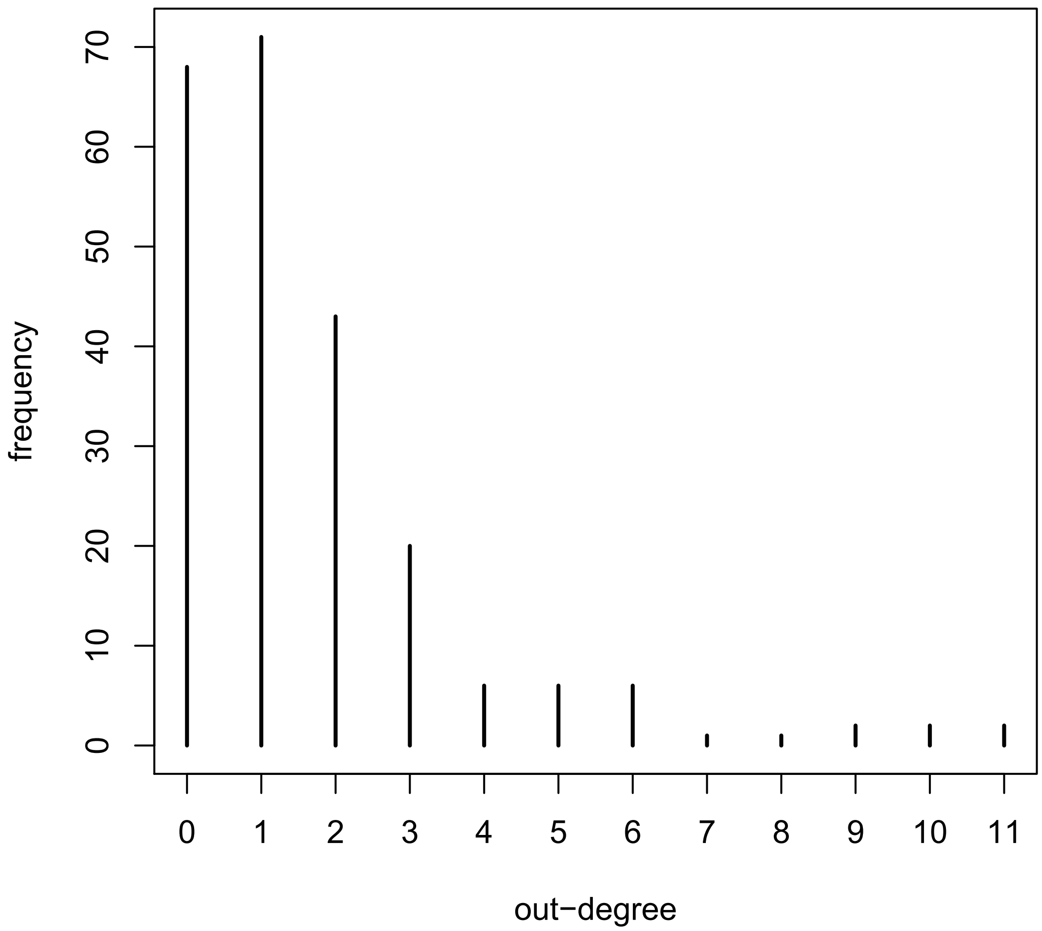

Additional Figure 9 – In-Degree Distribution of the HRG-Induced Gene Network

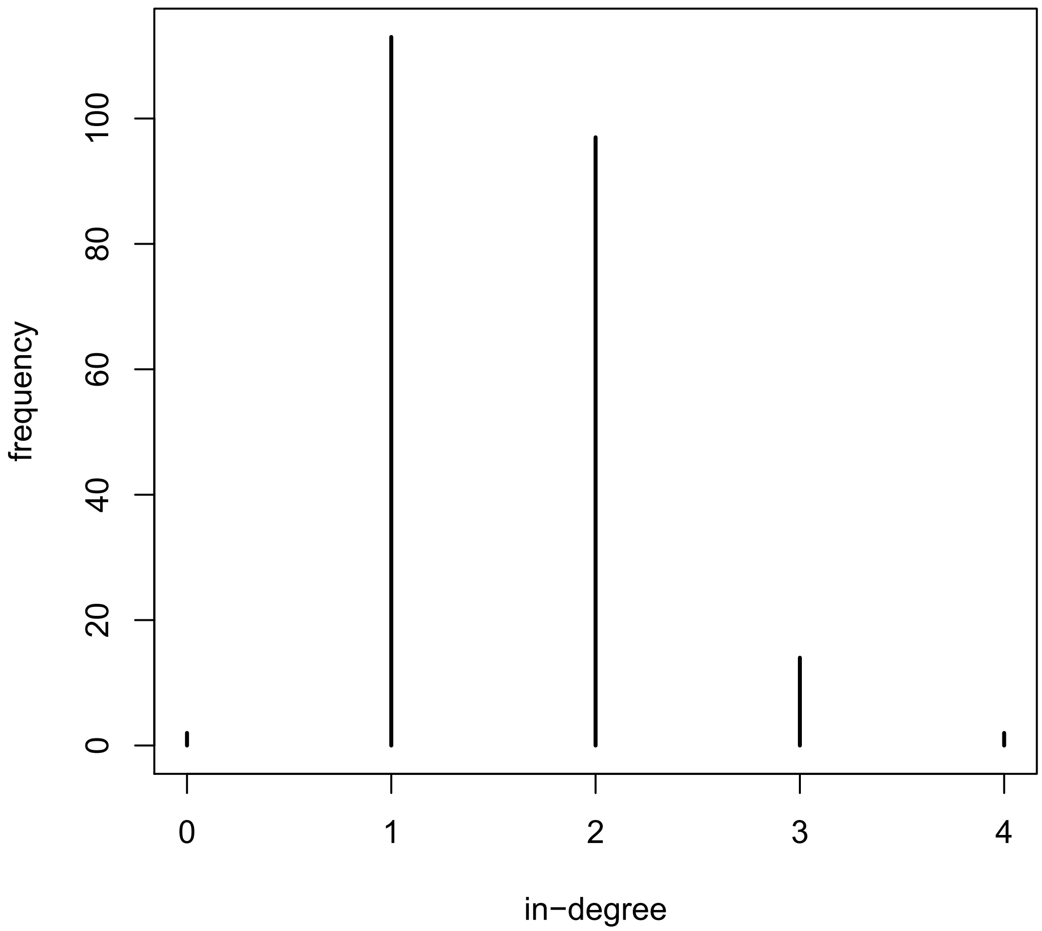

Additional Figure 10 – Out-Degree Distribution of the HRG-Induced Gene Network

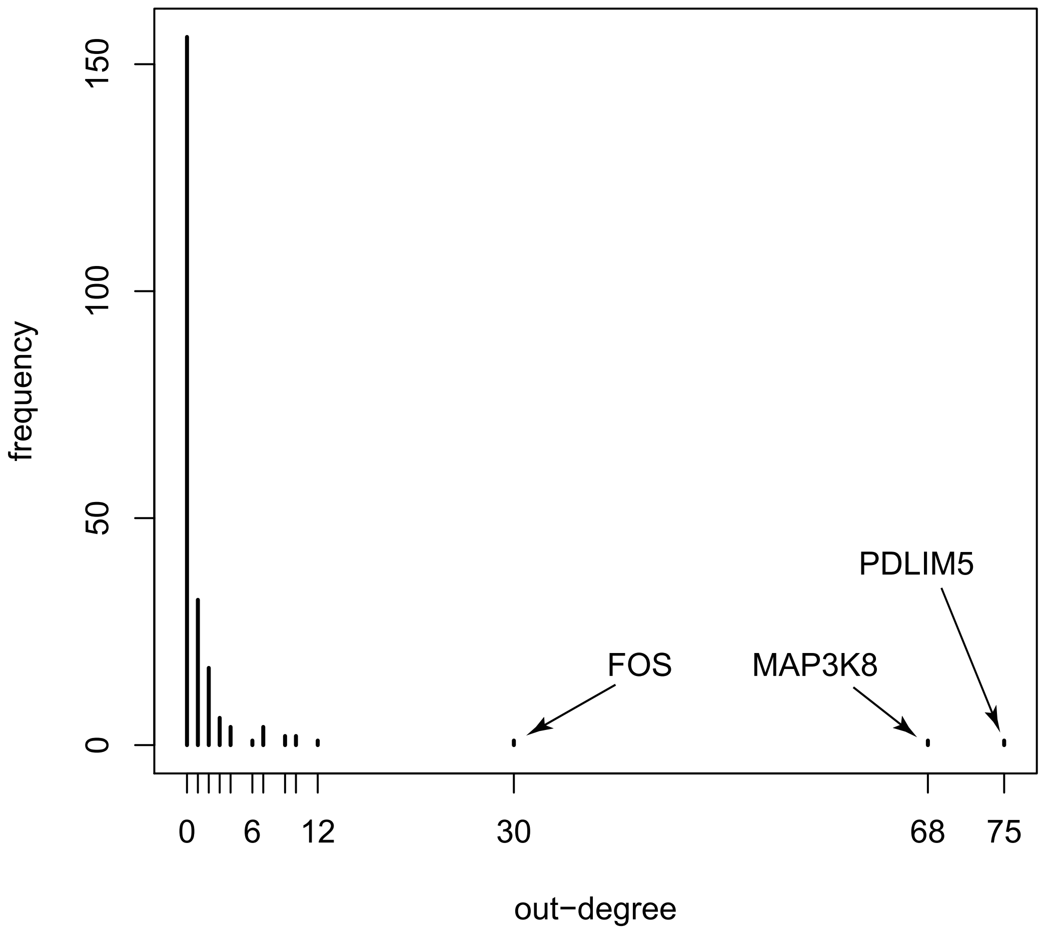

Supplement: Additional file 3 — In-degree and out-degree distributions of the EGF- and HRG-induced gene networks. This file includes Additional Figures 7 and 8 that describe the in-degree distribution and the out-degree distribution of the EGF-induced gene network, and Additional Figures 9 and 10 that describe the in-degree distribution and the out-degree distribution of the HRG-induced gene network, respectively. [file 1752-0509-3-41-S3.pdf]
